# Multiple polypoid colonic metastases from rectal adenocarcinoma with signet ring cells features: a case report

**DOI:** 10.1186/s12876-020-01493-8

**Published:** 2020-10-14

**Authors:** Yunlong Wu, Jiaolin Zhou, Tongtong Liu, Lai Xu, Yi Xiao

**Affiliations:** 1 Department of General Surgery, Peking Union Medical College Hospital, Peking Union Medical College, Chinese Academy of Medical Sciences, No. 1 Shuai Fu Yuan, Dong Cheng District, Beijing, 100730 China; 2 Department of Radiology, Beijing Chao-Yang Hospital, Capital Medical University, Beijing, 100020 China

**Keywords:** Rectal cancer, Signet ring cell carcinoma, Metastasis, Endoscopy, Imaging studies

## Abstract

**Background:**

Multiple polypoid colonic metastases are very rare which mainly originated from gastric carcinoma or melanoma. For rectal cancers, liver, lung and peritoneum are the most common metastatic sites. Here we present an unusual case with rectal adenocarcinoma and metachronous multiple colonic polypoid metastases.

**Case presentation:**

A 53-year-old man who underwent radical resection for rectal cancer 2 years ago was admitted to our department for an elevation of CEA level of 18.4 ng/ml. Colonoscopy revealed ten ivory rubbery colonic polypoid lesions (about 5 mm in diameters) in the large bowel which were confirmed as signet ring cell carcinomas (SRCC) by biopsy, but full-body contrast enhanced CT and PET-CT showed no other suspicious lesion. Seven weeks later, a laparoscopic total colectomy was performed and more than 50 polypoid lesions were observed throughout the mucosal surface of the large intestine which were confirmed as metastatic SRCC by postoperative pathological examination. All the 34 paracolic lymph nodes retrieved were involved. After 4 months, diffuse abdominopelvic and multiple bone metastases were identified by CT and the patient died of the disease 1 month later.

**Conclusion:**

Here we present an unusual case of multiple colonic polypoid metastases of rectal adenocarcinoma. For SRCC that is prone to have disseminated micrometastases, colonic ‘polyps’ may be the early noticeable sign of undetectable and extensive tumor spread. Instead of surgical resection of ‘the confined disease in colon’, systemic treatment maybe a more appropriate choice.

## Background

Multiple polypoid colonic metastases from other distant gastrointestinal carcinomas are extremely rare, only a few cases have been reported in the literatures [1–7]. Among these cases, colonic metastases were mainly originated from gastric carcinoma or melanoma, with signet ring cell features being the most common histological type. Signet ring cell carcinoma (SRCC) is a rare subtype of colorectal cancer, accounting for about 1% of all rectal cancers [8, 9]. Patients with SRCCs often present with distinct clinical features and poor prognosis [10, 11].

The most common sites of metastases from rectal cancer are the liver, lung, and peritoneum. Here, we report an unusual rectal cancer case with metachronous multiple colonic polypoid metastases.

## Case presentation

A 53-year-old man with progressive abdominal pain and distention was admitted to our tertiary care center on October 15, 2013. Colonoscopy revealed a rigid circumferential neoplasm in the rectum about 8–9 cm from the anal verge, with an increased CEA level of 10.4 ng/ml. In addition, two sessile polys (2–3 mm) in descending colon were also found and removed by forceps during colonoscopy, which were pathologically confirmed as adenomas. MRI showed a circumferential thickening of the bowel wall (maximum thickness, 14 mm), suggesting the diagnosis of malignant tumor (cT3N0Mx). After three times of fruitless biopsies, which invariably revealing “inflammation”, a transrectal incisional biopsy under the general anesthesia was performed and poorly differentiated adenocarcinoma was diagnosed. The patient received the neoadjuvant chemoradiotherapy (long-term radiation of 50 Gy/25f with concomitant capecitabine) and followed by the radical abdominoperineal resection surgery. The postoperative pathology showed poorly-differentiated adenocarcinoma with signet ring cell features (staged ypT3N1b). Thereafter, the patient received six cycles of adjuvant single-agent chemotherapy (capecitabine), owing to his refusal of the recommended intensified regimens.

**Fig. 1:**
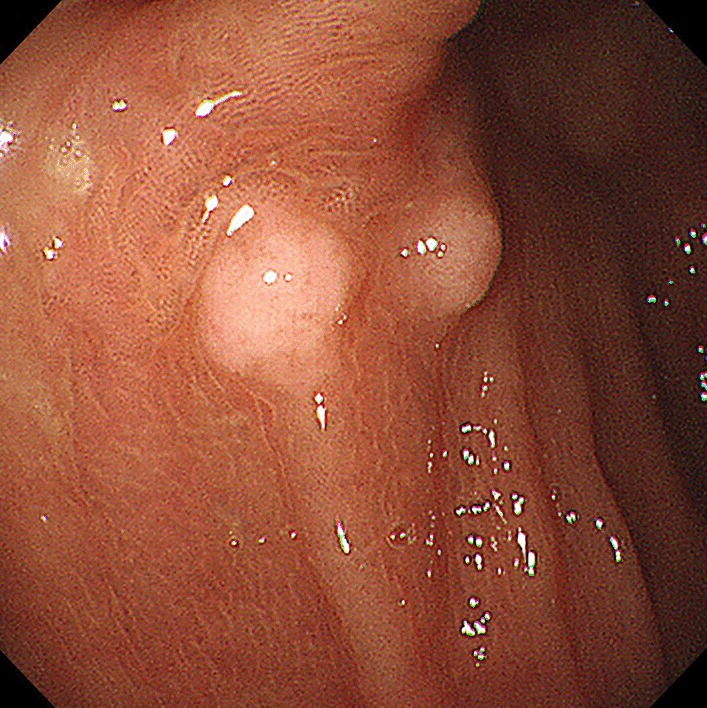
Colonoscopy images showing multiple “polyps” throughout colon

**Fig. 2:**
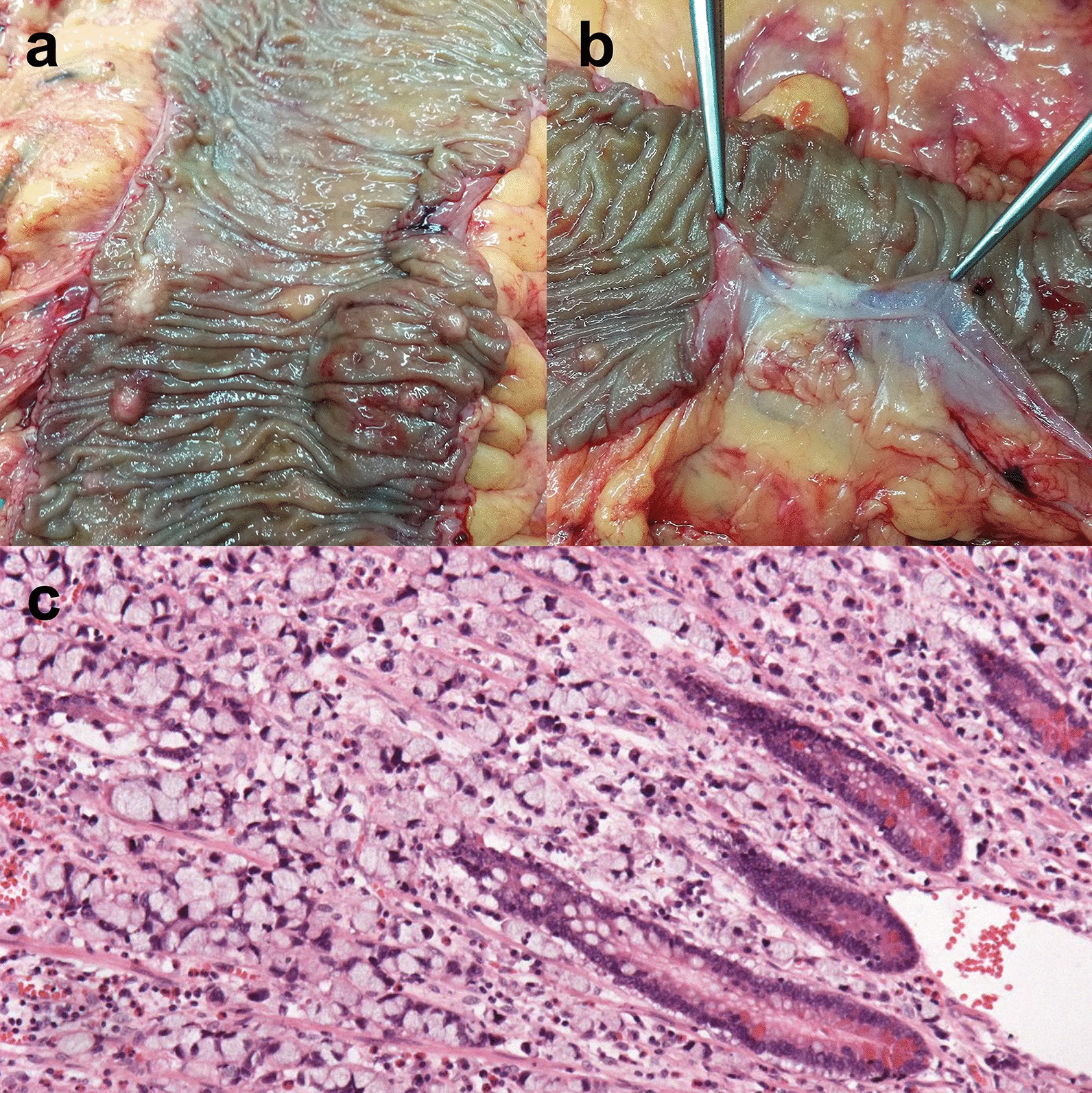
The resection of entire colon showed multiple white polypoid lesions (**a**, **b**), and histopathological examination result revealing signet ring cells (**c**, hematoxylin and eosin staining; magnification, × 100)

During regular follow-up, the patient was diagnosed with thrombocythemia and began to take hydroxyurea 1 g twice a day. Two years after surgery, the CEA level increased significantly from 2.9 to 18.4 (ng/mL). Colonoscopy revealed about ten polypoid lesions (about 5 mm in diameter), and the biopsies proved to be SRCC (Fig. 1). Imaging examinations including full-body contrast enhanced CT, PET-CT and gastroscope were then performed, with no other primary or metastatic foci being identified. The patient refused further systemic chemotherapy, but insisted on surgery. Two months later, a laparoscopic total colectomy with permanent ileostomy was performed. By examining the gross specimen, more than 50 polypoid lesions were scattered throughout the colonic wall, ranging from 2 to 10 mm in diameter (Fig. 2a). Pathology showed multifocal signet ring cell carcinomas with some lesions involving the whole layer of colonic wall (Fig. 2b–c), as well as a large number of metastatic nodules throughout the mesocolon. All the 34 paracolic lymph nodes retrieved were involved. Immunohistochemically, the metastatic tumors were CEA (focal+), CK7 (−), CK20 (+), MUC2 (+), MUC5AC (−), MUC6 (−), E-Cadherin (+), MLH-1 (+), MSH-2 (+), MSH-6 (+), PMS-2 (+), Ki-67 index: 70%. After 4 months, diffuse abdominopelvic and multiple bone metastases were detected through the CT and the patient died of the disease 1 month later.

In additional investigation by targeted next generation sequencing using a 1021-gene panel, somatic mutations of *ALK, EPHB2, ERBB4, GRIN2A, PTPRD, TP53* were detected in tumor tissue.

Informed consent documents were provided by patients’ surrogates.

## Discussion and conclusion

Polypoid colonic metastases are extremely uncommon, which has been previously reported less than 10 cases [1]. In this case, the pathologic examination demonstrated the same histological type between the colonic polypoid lesions and the original rectal cancer. Also, other malignancies including the gastric cancer were excluded by the gastroscope and PET-CT. Besides, the immunohistochemical exam of the colonic metastases showed CK7^−^/CK20^+^, which indicated the gastrointestinal origin [12, 13]. Therefore, we conclude that this is an extremely rare case of multiple polypoid colonic metastases from the rectal carcinoma with signet ring cell features.

Patients with SRCC often have distinct clinical characteristics and poor outcomes [11]. Evidence indicated that in comparison with well to moderately differentiated colorectal adenocarcinoma, SRCC was prone to have widespread peritoneal metastases, instead of common liver or lung metastases [14, 15]. SRCC was believed to be associated with distinctive molecular features. Study have shown that tumor cells of SRCC have impaired expression of adhesion molecules including E-cadherin and β-catenin [16], which may attribute to the loose appearance and aggressive behavior. In this case, we found disseminated macroscopic metastases confined to the mesocolon and bowel wall during surgery. Whether these lesions spread via uncommon route, such as hematogenous or lymphatic spread through the submucosal or mesenteric vasculatures, is still elusive.

As the colonic polypoid metastases implied the wide spread of the disease, most of the previous cases were treated with a non-operative approach using systemic chemotherapy [1, 5, 6] or best supportive care [7], and the outcomes were diverse and poor.

In 2015, Hugen et al. [17] found that adjuvant chemotherapy could improve the survival of stage III colorectal signet ring cell carcinoma. A recent study also revealed that, compared with surgery alone, chemotherapy alone or combined with surgery may further improve the prognosis of SRCC patients with peritoneum metastases [18]. In this case, unfortunately, the unusual metastatic pattern and the false-negative imaging findings misguided the surgeons to perform an unbeneficial colectomy surgery. We suppose that the feature of micrometastases in SRCC make it difficult to identify the wide spread of the disease by imaging examinations, even in patients with heavy tumor burden.

In summary, the presence of multiple polypoid colonic metastases may represent a very rare condition of extensively systemic spread of SRCC. Even without evidence of involvement of other organs, we think there is no role for colectomy in this condition. Systemic treatment with intensified chemotherapy and new targeted therapies may bring a glimmer of hope for patients with such refractory cancer.

## Data Availability

All data generated or analyzed during this study are included in this current article.

## Abbreviations

SRCC: Signet ring cell carcinoma
CT: Computed tomography
MRI: Magnetic resonance imaging
PET-CT: Positron emission tomography-computed tomography
CEA: Carcino-embryonic antigen
CK7: Cytokeratin 7
CK20: Cytokeratin 20
MUC2: Mucin 2
MUC5AC: Mucin 5AC
MUC6: Mucin6
MLH1: MutL homolog 1
MSH2: MutS homolog 2
MSH6: MutS homolog 6
PMS2: Postmeiotic segregation increased 2
